# Evaluating possible acute coronary syndrome in primary care: the value of signs, symptoms, and plasma heart-type fatty acid-binding protein (H-FABP). A diagnostic study

**DOI:** 10.3399/bjgpopen19X101652

**Published:** 2019-07-10

**Authors:** Robert TA Willemsen, Bjorn Winkens, Bas LJH Kietselaer, Agnieszka Smolinska, Frank Buntinx, Jan FC Glatz, Geert-Jan Dinant

**Affiliations:** 1 General Practitioner, PhD, Department of Family Medicine, Maastricht University, Maastricht, the Netherlands; 2 Assistant Professor, Department of Methodology and Statistics, Maastricht University, Maastricht, the Netherlands; 3 Cardiologist, Department of Cardiology, Zuyderland Hospital, Heerlen, the Netherlands; 4 Assistant Professor, Department of Pharmacology & Toxicology, Maastricht University, Maastricht, the Netherlands; 5 Professor, Department of Public Health and Primary Care, Catholic University Leuven, Leuven, Belgium; 6 Professor, Department of Family Medicine, Maastricht University, Maastricht, the Netherlands; 7 Professor, Department of Genetics & Cell Biology, Maastricht University, Maastricht, the Netherlands; 8 Professor, Department of Family Medicine, Maastricht University, Maastricht, the Netherlands

**Keywords:** primary health care, chest pain, acute coronary syndrome, early diagnosis, point-of-care testing, biomarkers

## Abstract

**Background:**

Additional diagnostic means could be of added value when evaluating possible acute coronary syndrome (ACS) in primary care.

**Aim:**

To determine whether heart-type fatty acid-binding protein (H-FABP)-based point-of-care (POC) biomarker testing, embedded in a clinical decision rule (CDR), is helpful to the GP when evaluating possible ACS.

**Design & setting:**

A prospective, non-randomised, double-blinded, diagnostic derivation study was undertaken, with a delayed-type cross-sectional diagnostic model among GPs in the Netherlands and Belgium.

**Method:**

Signs and symptoms predicting acute myocardial infarction (AMI) or ACS were identified using both logistic regression analysis, and classification and regression trees (CART). Diagnostic values of the POC H-FABP test (cut-off value 4 ng/ml) alone and as part of a CDR were determined.

**Results:**

A total of 303 participants (48.8% male) with chest pain or discomfort who had consulted a GP were enrolled. ACS was found in 32 (10.6%) of these 303 patients. For ACS, sensitivity and negative predictive value (NPV) of the POC H-FABP test was 25.8% (95% confidence interval [CI] = 12.5 to 44.9) and 91.6% (95% CI = 87.6% to 94.5%), respectively. The area under the receiver operating curve of the optimal CDR was 0.78 for ACS.

**Conclusion:**

Sensitivity of the current H-FABP POC test (cut-off value 4 ng/ml) as a stand-alone test is poor, either owing to limitations of the marker or of the test device. Usability of a CDR derived from these results is doubtful: the number of ACS cases missed by the GP is reduced but, as a consequence, disproportionally more ACS-negative patients are referred.

## How this fits in

Biomarkers of myocardial cell damage are the cornerstone of ruling in and out ACS in secondary care and might play a role in triaging patients presenting with chest pain in primary care. However, sensitivity of a studied PoC H-FABP test (cut-off 4 ng/ml) as a stand-alone test appeared insufficient to support its use for ruling out ACS in primary care. A CDR was not of added value when compared with the current unaided clinical judgment of the GP. Future studies should focus on highly sensitive and adequately specific tests to improve triage of these patients.

## Introduction

### Background

In primary care, 1.26% of all consultations are about chest pain.^1^ Among these patients, cases of ACS are largely outnumbered by less severe causes of chest pain.^1–5^ Yet, to avoid missing ACS, the GP's threshold to refer patients with chest pain is low.^1,5–7^ To reduce ACS-negative referrals in primary care and thereby diminish costs and patient discomfort — without a rise in missed cases of ACS — a CDR based on a clinical score of signs, symptoms, and POC biomarker testing detecting myocardial cell damage (such as high-sensitive cardiac troponin [hs-cTn] or H-FABP) is desirable.^3,8–17^ A potential role for H-FABP POC testing was earlier found using a cut-off value of 4 ng/ml, in an emergency department setting.^18^

### Objectives

This study aimed to:

1. derive signs and symptoms that have diagnostic value in a CDR to predict or exclude ACS and AMI (stage 2 of the 6 major stages in the development and testing of a CDR, as defined by Stiell and Wells, or step 2 and 3 as defined by Van den Bruel *et al*).^19,20^
2. assess the diagnostic value of a PoC H-FABP test for the outcomes: (1) life-threatening disease; (2) ACS (that is, AMI or unstable angina [UA]); and (3) AMI alone.
3. assess the diagnostic value of a CDR, including signs, symptoms, and POC H-FABP testing for these outcomes (internal validation).

For an extended background of this study and full details on the methodology, see the authors' previously published research protocol and case reports.^21,22^

## Method

### Study design

A prospective, non-randomised, double-blinded, diagnostic derivation study with a delayed-type cross-sectional diagnostic model was performed.^23^

### Recruitment: inclusion and exclusion criteria

**Figure 1.**
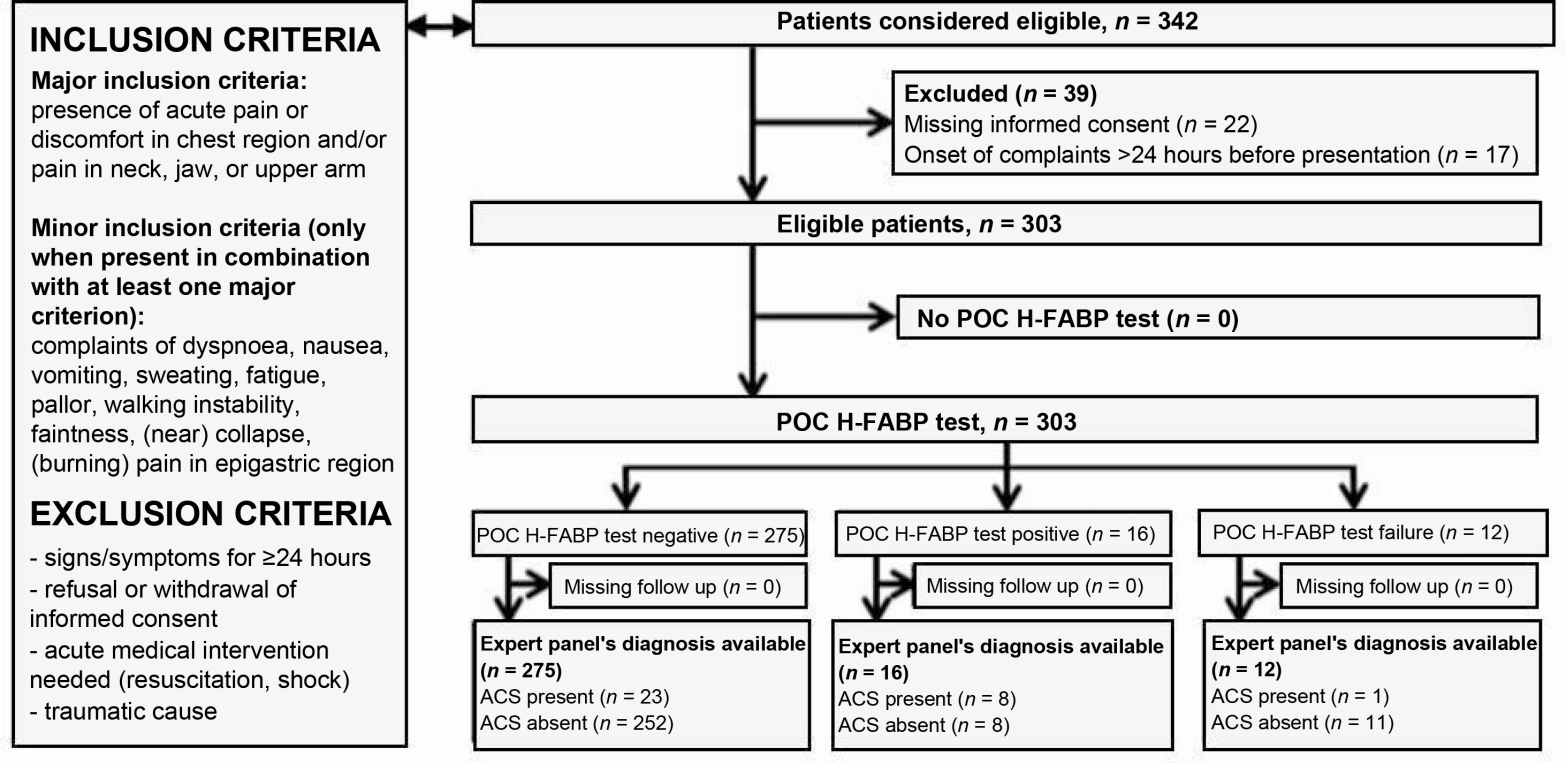
Study flow chart showing inclusion and exclusion criteria, and eligible participants with numbers of main outcome (ACS). ACS = acute coronary syndrome. H-FABP= heart-type fatty acid-binding protein. POC = point of care.

It was proposed to let 60 GPs in the southeast of the Netherlands and the northeast of Belgium include 10 patients with chest pain each. For inclusion criteria, see Figure 1. However, GPs faced difficulties in performing the necessary study procedures (for example, informed consent, venous sampling) while being urgently consulted for chest pain of recent onset. Therefore, the study was extended to several out-of-hours (OOH) primary care facilities where incidence of chest pain is higher and specialised nurses are available to aid with study procedures.

### Data collection

After obtaining verbal informed consent (for patients in Belgium) or a short version of the written informed consent (for patients in the Netherlands), the standardised case report form was completed by the GP by registration of history, physical examination, electrocardiogram (ECG), if performed, and the presumptive diagnosis together with the original decision whether or not to refer to a cardiologist. Then, a POC H-FABP test with a cut-off value of 4 ng/ml (manufactured by FABPulous BV) was performed. Subsequently, the original GP's decision was followed, unless a decision not to refer a patient was followed by a positive H-FABP test. In those cases, GPs were instructed to overrule their original decision and refer the patient, based on the positive test result. Patients who were not referred to secondary care were instructed to have a venous blood sample drawn for measurement of hs-cTn (as a gold standard to rule out AMI in non-referred patients), creatinine, and estimated glomerular filtration rate, within the interval between 3 hours and 3 days after onset of complaints. Within 7 days of the initial consultation, patients (in both Belgium and the Netherlands) were given the opportunity to complete written informed consent after having read an information letter at a more convenient moment. In the event of refusal at this point, patients were excluded.

### Final diagnosis and study outcomes: life-threatening disease, AMI, and ACS

After 30 days, all available patient data were collected by the study team. Final diagnoses were established by an expert panel of one independent GP and one independent cardiologist. The expert panel was blinded to the PoC H-FABP test result. The expert panel had access to all other patient data, including troponin measurements. The final diagnosis was based on these results, including the gold standard troponin test results, if available. All outcome diagnoses were determined following current definitions of conditions as cardiac (ischaemic) disease, stable angina (SA), UA, AMI, and ACS, as extensively detailed in the authors' published research protocol.^21^ Diseases labelled as life-threatening were: ACS (that is, UA and AMI), acute heart failure, pulmonary embolism, aortic dissection, and sudden death. The main study outcomes were: life-threatening causes, ACS, and AMI.

### Statistical analysis

To assess the predictive parameters for the main outcomes, χ^2^ tests or Pearson correlation analyses of the data were used. Using multiple logistic regression analysis, variables significantly related to ACS and AMI were selected for inclusion in CDRs. Subsequently, several combinations of these parameters and the H-FABP test results were combined to formulate CDRs based on scoring systems with straight cut-off values to aid clinical usability. The diagnostic value of the CDR for the study outcomes of ACS and AMI, respectively, was assessed.^24,25^ Additionally, CART analysis was performed to identify predicting factors and to formulate a CDR. The CART model was validated using bootstrap aggregation. Within this procedure, two-thirds of the samples were randomly chosen with replacement to build the classification model, while the remaining one-third of the samples were used to internally validate the predictive ability of the CART model. The entire procedure was repeated 1000 times to ensure that most of the samples were selected for training and validating the CART model. CART is based on binary recursive partitioning. The advantages of CART include the fact that it is inherently non-parametric and that the interpretation of results in the trees is plain, making a straightforward translation to clinical practice obvious.^26^ Data were processed using Matlab (version R2017b, for the CART analysis) and IBM SPSS Statistics for Windows (version 23). *P* values ≤0.05 were considered statistically significant.

### Data management

Handling of the personal data complied with the Dutch Personal Data Protection Act and Belgian privacy legislation. The study is in agreement with the current version of the WMA Declaration of Helsinki and is in accordance with the Dutch Medical Research Involving Human Subjects Act (WMO).^27^

## Results

### Patient characteristics

A total of 342 patients were selected for inclusion by the participating GPs, and 39 patients were excluded (see Figure 1). Thus, 303 patients were included in the study. Patient characteristics are shown in Table 1.

**Table 1.** Patient and population characteristics of study population (*n* = 303)

Variable	*n* (%)	Remarks
Sex	Male	148 (48.8)	–
	Female	155 (51.2)	
Age, years	≤30	15 (5.0)	Mean age, years (range): 58.3 (17–100)
	31–40	25 (8.3)	
	41–50	47 (15.5)	
	51–60	78 (25.7)	
	61–70	74 (24.4)	
	71–80	43 (14.2)	
	>80	21 (6.9)	
Type of contact(missing data *n* = 1)	Consultation, daytime, own GP	139 (46.0)	150 patients (49.7%) seen by their own GP during office hours, 152 patients (50.3%) seen by GP on call at OOH service
	Home visit, daytime, own GP	11 (3.6)	
	Consultation, OOH service	146 (48.3)	
	Home visit, OOH service	6 (2.0)	
History	No DM2 or prior CVD	179 (59.1)	–
	DM2	22 (7.3)	
	Prior CVD	90 (29.7)	
	DM2 and prior CVD	12 (4.0)	
Duration of complaints at presentation, hours(missing data *n* = 6)	0–1	32 (10.8)	–
	2–3	83 (27.9)	
	4–6	62 (20.9)	
	7–12	52 (17.5)	
	13–24	68 (22.9)	
eGFR, ml/min(missing data *n* = 49)	≤30	3 (1.2)	–
	31–60	42 (16.5)	
	>60	09 (82.3)	
ECG performed(missing data *n* = 1)	No	50 (16.6)	–
	Yes, no abnormalities	152 (50.3)	
	Yes, with abnormalities	100 (33.1)	
Referral policy	No referral	167 (55.1)	Overall referral percentage: 44.9% Overall direct referral percentage: 37.0% Overall referral percentage at later time: 7.9%
	Direct referral on presentation to cardiologist	107 (35.3)	
	Direct referral on presentation, other	5 (1.7)	
	Referral at later time during follow-up, cardiologist	20 (6.6)	
	Referral at later time during follow-up, other	4 (1.3)	
Troponin available	Troponin available (all patients)	267 (88.1)	
	Troponin available (non-referred patients)	171 (87.2)	
	Troponin available (referred patients)	96 (89.7)	

CVD = cardiovascular disease. DM2 = type 2 diabetes mellitus. ECG = electrocardiogram. eGFR = estimated glomerular filtration rate. OOH service = out-of-hours service.

### Final diagnoses

After 30 days, ACS was the final diagnosis in 32 (10.6%) of the included patients, and other life-threatening disease was found in three additional patients (all diagnosed with heart failure). Two patients (0.7%) died shortly after assessment by the GP. Of all patients with chest pain, 75.2% suffered from non-cardiac disease. All final diagnoses, as assessed by the expert panel, are available from the authors on request.

### Study objective 1: diagnostic value of the POC H-FABP test

Table 2 shows diagnostic parameters for all patients, patients presenting within 3 hours after onset of complaints, and patients presenting 3–24 hours after onset of complaints for several outcomes of the POC H-FABP as a stand-alone test, without considering additional signs and symptoms. Extended data are available from the authors on request. For ACS, sensitivity of the POC H-FABP test was 25.8% (36.8% for the subgroup of patients with complaints for more than 3 hours). NPV for ACS was 91.6%.

**Table 2.** Diagnostic parameters of POC H-FABP as a stand-alone test

	Outcome: ACS or other life-threatening disease	Outcome: ACS	Outcome: AMI	Outcome: NSTEMI
**Interval, onset complaints to presentation, hours**	**0–** **24**	**0–** **3**	**3–** **24**	**0–** **24**	**0–** **3**	**3–** **24**	**0–** **24**	**0–** **3**	**3–** **24**	**0–** **24**	**0–** **3**	**3–** **24**
**Patients, *n***	291^a^	106^b^	179^b^	291^a^	106^b^	179^b^	291^a^	106^b^	179^b^	291^a^	106^b^	179^b^
**Sens H-FABP POCT, % (95% CI**)	25.7 (13.1 to 43.6)	8.3 (0.44 to 40.2)	36.4 (18.0 to 59.2)	25.8 (12.5 to 44.9)	9.1 (4.8 to 42.9)	36.8 (17.2 to 61.4)	26.9 (12.4 to 48.1)	10.0 (0.52 to 45.9)	40.0 (17.5 to 67.1)	18.9 (5.0 to 46.3)	0 (0 to48.3)	33.3 (9.0 to 69.1)
**Spec H-FABP POCT (95% CI**)	97.3 (94.2 to 98.8)	98.9 (93.4 to 99.9)	96.2 (91.5 to 98.4)	96.9 (93.8 to 98.6)	98.9 (93.4 to 99.9)	95.6 (90.8 to 98.1)	96.6 (93.4 to 98.3)	99.0 (93.5 to 99.9)	95.1 (90.3 to 97.7)	95.3 (91.9 to 97.4)	98.0 (92.3 to 99.7)	93.5 (88.4 to 96.6)
**NPV H-FABP POCT, % (95% CI**)	90.5 (86.3 to 93.6)	9.4 (81.5 to 94.3)	91.5 (85.9 to 95.1)	1.6 (87.6 to 94.5)	90.4 (82.6 to 95.0)	92.7 (87.4 to 96.0)	93.1 (89.2 to 95.7)	91.3 (83.8 to 95.7)	94.5 (89.6 to 97.3)	95.3 (91.9 to 97.4)	94.2 (87.4 to 97.6)	96.4 (91.9 to 98.5)
**PPV H-FABP PoCT, % (95% CI**)	56.3 (30.6 to 79.2)	50.0 (26.7 to 97.3)	57.1 (29.6 to 81.2)	50.0 (25.5 to 74.5)	50.0 (26.7 to 97.3)	50.0 (24.0 to 76.0)	43.8 (20.8 to 69.4)	50.0 (26.7 to 97.3)	42.9 (18.8 to 70.4)	18.9 (5.0 to 46.3)	0 (0 to 80.2)	21.4 (5.7 to 51.2)

Sensitivity, specificity, negative and positive predictive values of POC H-FABP for all patients, patients presenting within 3 hours of onset of complaints, and patients presenting 3–24 hours after onset of complaints for several outcomes, namely: (1) life-threatening disease (that is, composite of acute heart failure, pulmonary embolism, aortic dissection, acute death, ACS); (2) ACS (that is, UA, NSTEMI, STEMI); (3) AMI (that is, NSTEMI and STEMI); and (4) NSTEMI only.

^a^12 test failures among 303 included patients.

^b^ In six cases, patients presented within 24 hours after onset of complaints; however, registration of duration of complaints was incomplete. These patients are not included in the tables representing subgroups of patients presenting with chest pain within 3 hours or 3–24 hours after onset of complaints.

ACS = acute coronary syndrome. AMI = acute myocardial infarction. CI = confidence intervals. H-FABP = heart-type fatty acid-binding protein. NPV = negative predictive value. NSTEMI = non-ST elevated myocardial infarction. POC = point of care. PPV = positive predictive value. Sens = sensitivity. Spec = specificity. STEMI = ST-elevated myocardial infarction. UA = unstable angina.

Test failure was reported in 12 out of 303 cases (4.0%). The POC H-FABP test was positive in 16 cases (5.3%), as shown in Figure 1. In five of these cases, the GP had no intention to refer before testing. Following the study protocol, all five patients were referred. In one of these cases, an ACS was found as a final diagnosis. In the remaining four cases, no cardiac diagnosis was identified. Additional comments and details on false positive and false negative test results are available from the authors on request.

### Study objective 2: derivation of a CDR, and univariable and multivariable analyses

Correlations between signs and symptoms and outcome measures were tested in a bivariate analysis (available from the authors on request). For ACS, significant associations were found for age, absence of left-sided lateral chest pain, a feeling of pressure on the chest, pain in right arm or throat, palor or sweating on physical examination, several ECG-abnormalities, and a positive H-FABP PoC test. No significant influence was seen for the parameters 'duration of complaints', 'seen by own GP or seen at OOH service', 'sex', and 'history of cardiovascular disease (CVD)' or 'history of type two diabetes mellitus (DM2)'.

**Figure 2.**
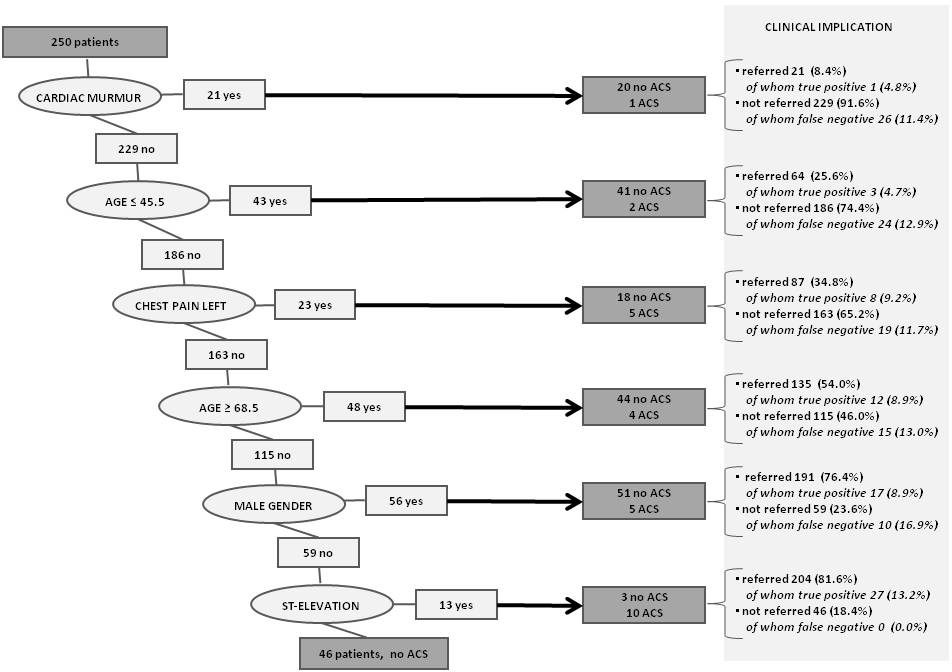
Classification tree for acute coronary syndrome. ACS = acute coronary syndrome.

In a multiple logistic regression analysis of all signs and symptoms that revealed significant predictive value in the univariable analysis, five highly significant predictors for ACS and AMI were found: ST-depressions, ST-elevations, dyspnoea, a feeling of pressure on the chest, and absence of left-sided lateral chest pain. CART analysis (classification model repeated 1000 times, bootstrap used to validate the model) revealed six successive predictors for ACS: cardiac murmur, age <45 years, left lateral chest pain, age ≥68.5 years, male sex, and ST-elevations (Figure 2). Different combinations of signs and symptoms (with and without POC H-FABP testing) were combined in possible CDRs. Sensitivity and specificity with 95% CIs of these combinations were calculated. CDR 1–4 were based on signs and symptoms derived from logistic regression analysis of the study data: in CDR 1, all relevant signs and symptoms found in logistic regression were combined with the POCT H-FABP result; in CDR 2, only signs and symptoms were included, excluding the POCT H-FABP result; CDR 3 is similar to CDR 1, excluding ECG findings (ECG equipment is not always available in primary care); and CDR 4 is based on relevant signs and symptoms without ECG and POCT H-FABP result. CDR 5 was based on CART analysis of the study data (Table 3).

**Table 3.** Sensitivity and specificity of possible clinical decision rules based on multivariable analyses of the study data (total patients with chest pain analysed, *n* = 303; total patients with ACS, *n* = 32 [10.6%]; total patients with AMI, *n* = 27 (8.9%])

		**Clinical judgment GP**	**CDR variant 1**	**CDR variant 2**	**CDR variant 3**	**CDR variant 4**	**CDR variant 5** ^a,c^
CDR based on…	ST-depressions	/	1	1	/	/	/
	ST-elevations	/	1	1	/	/	/
	Dyspnoea	/	1	1	1	1	/
	Feeling of pressure chest	/	1	1	1	1	/
	Absent lateral chest pain left	/	1	1	1	1	–
	Age ≤45.5, ≥68.5 years	/	/	/	/	/	/
	Male sex	/	/	/	/	/	/
	Cardiac murmur	/	/	/	/	/	/
	POCT H-FABP result positive	/	1	/	1	/	/
CDR and/or decision characteristics	Maximum score	/	6	5	4	3	/
	Considered negative if…	GP did not refer	≤1	≤1	≤1	≤1	/
	Patients with a negative versus positive score	191 negative, 112 positive	145 negative, 158 positive	147 negative, 156 positive	157 negative, 146 positive	161 negative, 142 positive	46 negative, 204 positive
Rule-out characteristics	Sens; NPV for ACS [95% CI]	Sens 75.0 [56.2 to 87.9]; NPV 95.8 [91.6 to 98.0]	Sens 87.5 [70.1 to 95.9]; NPV 97.2 [92.6 to 99.1]	Sens 81.2 [63.0 to 92.1]; NPV 95.9 [90.9 to 98.3]	Sens 78.1 [60.0 to 90.0]; NPV 95.5 [90.7 to 98.0]	Sens 68.8 [49.9 to 83.3]; NPV 93.8 [88.6 to 96.8]	Sens 100 [84.5 to 100]; NPV 100 [90.4 to 100]
	FNs for ACS, *n* (% of total)	8 (2.6)	4 (1.3)	6 (2.0)	7 (2.3)	10 (3.3)	0 (0.0)
	TNs for ACS, *n* (% of total)	183 (60.4)	141 (46.5)	141 (46.5)	150 (49.5)	151 (49.8)	46 (18.4)
	Sens; NPV for AMI [95% CI]	Sens 70.4 [49.7 to 85.5]; NPV 95.8 [91.6 to 98.0]	Sens 88.9 [69.7 to 97.1]; NPV 97.9 [93.6 to 99.5]	Sens 81.5 [61.3 to 93.0]; NPV 96.6 [91.8 to 98.7]	Sens 77.8 [57.3 to 90.6]; NPV 96.2 [91.5 to 98.4]	Sens 66.7 [46.0 to 82.8]; NPV 94.4 [89.3 to 97.2]	Sens 81.5 [61.3 to 93.0]; NPV 0 [0 to 53.7]
Rule-in characteristics	Spec; PPV for ACS [95% CI]	Spec 67.5 [61.6 to 73.0]; PPV 21.4 [14.5 to 30.4]	Spec 52.0 [45.9 to 58.1]; PPV 17.7 [12.3 to 24.8]	Spec 52.0 [45.9 to 58.1]; PPV 16.7 [11.4 to 23.7]	Spec 55.4 [49.2 to 61.3]; PPV 17.1 [11.6 to 24.4]	Spec 55.7 [49.6 to 61.7]; PPV 15.5 [10.2 to 22.7]	Spec 20.6 [15.6 to 26.7]; PPV 13.2 [9.1 to 18.8]
	FPs for ACS, *n* (% of total)	88 (29.0)	130 (42.9)	130 (42.9)	121 (39.9)	120 (39.6)	177 (70.8)
	TPs for ACS, *n* (% of total)	24 (7.9)	28 (9.2)	26 (8.6)	25 (8.3)	22 (7.3)	27 (10.8)
	Spec; PPV for AMI [95% CI]	Spec 66.3 [60.4 to 71.8]; PPV 17.0 [10.8 to 25.5]	Spec 51.4 [45.4 to 57.5]; PPV 15.2 [10.2 to 22.0]	Spec 51.4 [45.4 to 57.5]; PPV 14.1 [9.2 to 20.8]	Spec 54.7 (48.6 to 60.7]; PPV 14.4 [9.3 to 21.4]	Spec 55.1 (49.0 to 61.0]; PPV 12.7 [7.9 to 19.6]	Spec 0.0 (0.0 to 2.1]; PPV 9.0 [5.8 to 13.5]
Rule in or out^b^		Rule in, out ACS. Rule in, out AMI	Rule in, out ACS. Rule in, out AMI	Rule in, out ACS. Rule in, out AMI	Rule in, out ACS. Rule in, out AMI	Non-decisive	Rule out ACS

^a^Denominator in calculations for CDR variant 5 is 250 patients, since CART was initially performed in a subgroup of 53 patients and internally validated in a subgroup of the remaining 250 patients.

^b^A CDR is considered as possibly relevant for rule in when the pre-test probability (based on the prevalence in the study population) of presence of an AMI (8.9%), or ACS (10.6%), is below the lower margin of the 95% CI of the PPV of the CDR. A CDR is considered as potentially relevant for rule-out when the pre-test probability of absence of an AMI (91.1%), or ACS (89.4%), is below the lower margin of the 95% CI of the NPV.

^c^In addition to the predicting signs and symptoms derived from CART for ACS, two other symptoms were added to CDR variant 5 for AMI: systolic blood pressure ≥149 mmHg and bradycardia (heart rate <60/min), based on the CART analysis with AMI as an outcome.

ACS = acute coronary syndrome. AMI = acute myocardial infarction. CI = confidence interval. FNs = false negatives. FPs = false positives. H-FABP = heart-type fatty acid-binding protein. NPV = negative predictive value. NSTEMI = non-ST elevated myocardial infarction. POCT = point of care test. PPV = positive predictive value. Sens = sensitivity. Spec = specificity. SBP = systolic blood pressure. TNs = true negatives. TPs = true positives.

### Study objective 3: diagnostic value of the CDR

Rule-in and rule-out characteristics for the different CDRs and area under the receiver operating curves were calculated (Table 3). Possible relevance of a CDR in ruling in or ruling out ACS or AMI was defined as an increase in probability of presence (ruling in) or absence (ruling out) of disease after using the particular CDR. Area under receiver operating curve for ACS was highest for CDR 1 (0.78). NPV, positive predictive value (PPV), and sensitivity were comparable to regular care (that is, the GP's clinical judgment only). False negativity count for CDR 1 (four missed cases) was lower than the false negativity count of the GP's judgment only (eight missed cases). However, specificity of CDR 1 was lower than the specificity of the GP's clinical judgment, leading to a 47.7% increase in false positive test results and thus an increase in ACS-negative referrals. For CDR 5, based on CART analysis, an NPV of 100% was reached; however, referral rate owing to a positive result of the CDR would increase from 37.0% (GP's judgment) to 81.4%.

## Discussion

### Summary

The diagnostic value of a POC H-FABP test as a stand-alone test was poor: sensitivity of the test for ACS was 25.8%, leading to a high number of false negative test results. An algorithm of a feeling of pressure on the chest, absence of left-lateral chest pain, dyspnoea, ST depressions, ST elevations, and the H-FABP POC test result (CDR 1) predicted the outcome of ACS reasonably well (sensitivity 87.5%, NPV 97.2%). However, the gain as compared with a GP's clinical judgment was limited: the false negativity rate was 50.0% lower (absolute reduction of 1.3 percentage points) at a price of a considerable 47.7% increase in over-referral of ultimately ACS-negative cases (absolute increase of 13.9 percentage points). Moreover, using CDR 1 would lead to the necessity of performing a POC test and an ECG as part of the CDR in all patients with chest pain. CART analysis led to a CDR with a high NPV, of 100% (compared with a NPV of 95.8% based on a GP's clinical judgment), without the necessity of performing additional tests. However, the referral rate using this CDR would be 81.6%; more than twice as high as a GP's regular referral rate.

### Strengths and limitations

This study was performed in daily primary care. The H-FABP POC test was tested in a representative clinical situation. The incidence of ACS was 10.6%, comparable with previous findings in the literature, where incidences of 1.5% to 22% were described.^28–33^ The study team focused on deriving factors to be included in an algorithm of signs and symptoms combined with POC H-FABP testing predictive of ACS (stage 2 of the aforementioned stages of Stiell and Wells).^19^ All criteria for a methodologically correct stage 2 (definition of outcomes and predictor variables, generalisability of subject selection, several statistical and methodological demands) are present in this study, except the inter-observer reliability for the clinical findings, which is difficult to measure in an acute setting. External validation of the formulated CDRs has not yet been performed.

During the runtime of the study, problems were faced regarding inclusion of sufficient patients; GPs embraced the study with enthusiasm, but faced serious barriers when actually including patients. The fact that patients with chest pain are usually unannounced, and therefore not scheduled, leads to the overrunning of consultation hours. In addition, the possible life-threatening underlying disease seemed to limit inclusion into the study. Moreover, study procedures such as obtaining (temporary) written informed consent were seen as thresholds. As a result, extension of the study from day care in primary care to OOH services was necessary. Finally, although the aim was to include 600 patients, only 303 patients were included.

### Comparison with existing literature

In an earlier study, based on a POC H-FABP test with a cut-off value of 7 ng/ml, performance of the test in clinical situations was insufficient.^28^ The present study worked with a promising new POC H-FABP test with a cut-off value of 4 ng/ml, based on a clinical study with patients with chest pain in a secondary care emergency setting.^18^ However, performance of this POC H-FABP test as a stand-alone test was insufficient. Several causes could be underlying.

One possible factor that should be taken into account as a cause of absence of biomarker rise is the primary care spectrum tested in this study, where complaints are less typical and presentation is often early. This could possibly have led to a delayed measurable biomarker rise (underperformance of the marker). This is not in accordance with earlier research.^18^ A comparison between the H-FABP POC test result and the absolute H-FABP concentration (to be measured by ELISA in stored plasma samples obtained from a selection of patients) is planned. Including patients with UA — a usually biomarker-negative condition — possibly compromises the marker performance. However, subgroup analyses of only patients with AMI revealed similar results and, in primary care, ruling-out strategies should aim at ACS, not AMI alone. Additionally, the test signal could be insufficient; for example, the discolouration of the test could take place at higher cut-off values than the cut-off of 4 ng/ml (underperformance of the POC test). Test calibration by the manufacturer, however, suggests high accuracy of the test. Third, slight discolouration of the POC test might have been under-detected by care providers participating in the study (underperformance of the test operator) but, in the current study, the POC H-FABP performance as a clinical tool was being tested and if under-detection of the test was a problem, the test should be improved to augment usability. Moreover, participating caregivers never complained about the usability of the test during the study.

### Implications for research and practice

Neither the currently evaluated H-FABP POC test (FABPulous BV, cut-off value 4 ng/ml) as a stand-alone test, nor a CDR incorporating this H-FABP POC test is of added value when compared with the experienced GP's clinical judgment. Annually, a Dutch GP at least briefly suspects approximately 35 patients with chest pain of ACS.^1^ For every GP using CDR 1, one missed case of ACS would be prevented over 2 years. However, in the same 2 years, 70 ECGs and 70 POC tests would be necessary and 30, instead of the usual 20, ACS-negative patients would be referred to secondary care facilities to rule out ACS. Future studies should focus on: large primary care populations; fusing existing algorithms into a strong tool; (combinations of) biomarker POC tests, and proving cost-effectiveness of such diagnostic means.^19,33–36^ Thus, cases of ACS should be adequately detected and referral rates should decrease as compared with current practice, improving patient comfort and lowering cost to society.
